# Effects of Methylphenidate on performance of a practical pistol shooting task: a quantitative electroencephalography (qEEG) study

**DOI:** 10.1186/1755-7682-4-6

**Published:** 2011-02-04

**Authors:** Flávia Paes, Sergio Machado, Oscar Arias-Carrión, Clayton Amaral Domingues, Silmar Teixeira, Bruna Velasques, Marlo Cunha, Daniel Minc, Luis FH Basile, Henning Budde, Mauricio Cagy, Roberto Piedade, Scott Kerick, Manuel Menéndez-González, Stephen D Skaper, Braxton A Norwood, Pedro Ribeiro, Antonio Egídio Nardi

**Affiliations:** 1 Panic and Respiration Laboratory, Institute of Psychiatry, Federal University of Rio de Janeiro, Rio de Janeiro, RJ, Brazil; 2 Faculty of Psychology, Brazilian Institute of Medicine and Rehabilitation (IBMR), Rio de Janeiro, Brazil; 3 National Institute for Translational Medicine (INCT-TM); Rio de Janeiro, Brazil; 4 Brain Mapping and Sensory Motor Integration, Institute of Psychiatry of Federal University of Rio de Janeiro (IPUB/UFRJ), Brazil; 5 Institute of Applied Neuroscience (INA), Rio de Janeiro, Brazil; 6 Department of Neurology, Philipps University-Marburg, Baldingerstrasse D-35033 Marburg, Germany; 7 Division of Epidemiology and Biostatistic, Institute of Health Community, Federal Fluminense University (UFF), Rio de Janeiro, Brazil; 8 Escola de Aperfeiçoamento de Oficiais (EsAO - Ministério do Exército), Brazil; 9 School of Physical Education, Laboratory of Motor Behavior, Federal University of Vale do São Francisco (UNIVASF) - Pernambuco, Brazil; 10 Division of Neurosurgery of the University of São Paulo Medical School, Brazil; 11 Laboratory of Psychophysiology, UMESP, Brazil; 12 Department of Movement and Training Science, Institute of Sport Science, Humboldt University Berlin, Germany; 13 U.S. Army Research Laboratory, Aberdeen Proving Ground, MD, USA; 14 Unit of Neurology, Hospital Alvarez-Buylla, Mieres, Spain; 15 Department of Pharmacology and Anesthesiology, University of Padova, Italy; 16 Department of Neurology & Epilepsy Center Hessen, Philipps University-Marburg, Baldingerstrasse D-35033 Marburg, Germany; 17 School of Physical Education, Bioscience Department (EEFD/UFRJ), Brazil

## Abstract

**Background:**

The present study examined absolute alpha power using quantitative electroencephalogram (qEEG) in bilateral temporal and parietal cortices in novice soldiers under the influence of methylphenidate (MPH) during the preparatory aiming period in a practical pistol-shooting task. We anticipated higher bi-hemispheric cortical activation in the preparatory period relative to pre-shot baseline in the methylphenidate group when compared with the control group because methylphenidate has been shown to enhance task-related cognitive functions.

**Methods:**

Twenty healthy, novice soldiers were equally distributed in control (CG; n = 10) and MPH groups 10 mg (MG; n = 10) using a randomized, double blind design. Subjects performed a pistol-shooting task while electroencephalographic activity was acquired.

**Results:**

We found main effects for group and practice blocks on behavioral measures, and interactions between group and phases on electroencephalographic measures for the electrodes T3, T4, P3 and P4. Regarding the behavioral measures, the MPH group demonstrated significantly poorer in shooting performance when compared with the control and, in addition, significant increases in the scores over practice blocks were found on both groups. In addition, regarding the electroencephalographic data, we observed a significant increase in alpha power over practice blocks, but alpha power was significantly lower for the MPH group when compared with the placebo group. Moreover, we observed a significant decrease in alpha power in electrodes T4 and P4 during PTM.

**Conclusion:**

Although we found no correlation between behavioral and EEG data, our findings show that MPH did not prevent the learning of the task in healthy subjects. However, during the practice blocks (PBs) it also did not favor the performance when compared with control group performance. It seems that the CNS effects of MPH demanded an initial readjustment period of integrated operations relative to the sensorimotor system. In other words, MPH seems to provoke a period of initial instability due to a possible modulation in neural activity, which can be explained by lower levels of alpha power (i.e., higher cortical activity). However, after the end of the PB_1_ a new stabilization was established in neural circuits, due to repetition of the task, resulting higher cortical activity during the task. In conclusion, MPH group performance was not initially superior to that of the control group, but eventually exceeded it, albeit without achieving statistical significance.

## Introduction

The integration of sensory information, the performance of cognitive operations, and the achievement of motor control are important processes that underlie brain function during the execution of a complex task. In these processes, the central nervous system (CNS) integrates information coming from multiple sensory channels, allowing the performance of specific goal-directed tasks [1]. During the initial stage of learning, the main requirement is the establishment of perceived sensory information with correct motor commands [2,3]. In this manner, subjects have to attend to sensory information to make a decision about which action will be triggered, and if feedback is provided, subjects must commit the perceived response to memory. This novel establishment involves an arbitrary sensorimotor association, similar to learning by trial and error, strictly related to attention, decision, and movement selection, as well as sensory feedback processing and working memory [4].

Regarding the implementation of a shooting task, we selected target shooting because it is an ecologically valid cognition-demanding task and its brain dynamics have been studied using electroencephalography (EEG) (for review see [5]). Many studies have examined the effects of cognitive load and learning on shooting performance [6-8], however, they did not investigate the effects of psychostimulant drugs, methylphenidate (MPH) for example, on performance of this type of task.

MPH belongs to the piperidine class of compounds and increases the levels of dopamine and norepinephrine in the brain through reuptake inhibition of the monoamine transporters [9]. Dopamine, a neurotransmitter, plays a role in feelings of pleasure and is naturally released in rewarding experiences [2,3]. Dopamine decreases "background firing" rates and increases the signal to noise ratio in target neurons by increasing dopamine levels in the brain [9]. As a result, the drug may improve attention and decrease distractibility in activities that normally do not hold the attention of children with attention deficit hyperactivity disorder (ADHD) [9].

Indeed, neuroimaging studies exploring the neural correlates multiple memory systems interact during learning have implicated dopaminergic pathways [10]. These results suggest that distinct human memory systems operate in parallel during probabilistic learning, and may act synergistically particularly when a violation of expectation occurs, to jointly contribute to learning and decision making [2,3]. On the other hand, several studies have demonstrated quantitative EEG (qEEG) changes after ingestion of MPH while investigating the ergogenic mechanisms of these drugs in order to better understand cerebral function [11-18].

MPH is used to treat ADHD and has been extensively misused, especially by college students as a "study aid" in order to improve their cognitive functions [19]. The findings involving MPH influence on cognition function are more diverse than those described for other drugs such as amphetamine, showing less consistency among outcomes [20]. Some studies do not show that MPH improves learning acquisition or that it improves other cognitive process, such as reversal learning or 'planning' [21-23]. However, an interesting recent study by Izquierdo et al. [24] suggests that MPH might promote recall at longer time intervals - but only in situations where performance is already impaired, such as in age-dependent memory decline. In effect, this evidence suggests that MPH does not drastically improve the formation of new memory in health people, but that it may facilitate long-term retention if taken after information has been acquired. Furthermore, the effect of MPH on other types of cognitive ability, specifically on tasks that require the subject to learn about relationships among stimuli and responses, does not seem to be very beneficial [25].

The precise effects of MPH on cognitive functions as measured by quantitative EEG remain unclear despite EEG's sensitivity in identifying functional changes produced by an exogeneous substance [26]. Therefore, the investigation of the effects of MPH on cortical dynamics and motor behavior is essential for advancing our understanding of the effects of cognition-altering pharmacological agents. With this in mind, the present study examined absolute alpha power in the left and right temporal and parietal cortices in novice soldiers receiving 10 mg MPH during the preparatory aiming period prior to the trigger pull in a practical pistol shooting task. Spectral features of the EEG in the alpha (8-12 Hz) band are sensitive to variations in perception, cognition or motor action [27]. Alpha activity reflects a form of cortical idling or deactivation [28-30], with its amplitude inversely related to cortical activation during perceptual, cognitive, and motor processes. Several studies demonstrated that the temporal and parietal cortices are involved in the transmission of multimodal sensory information [31,32]. Moreover, the parietal lobe integrates sensory information from different modalities and plays important roles in integrating sensory information from various parts of the body, in the manipulation of objects, as well as in attention and visuospatial processing (i.e. spatial sense and navigation) [33]. Therefore, we expected higher bi-hemispheric cortical activation in the preparatory period, relative to pre-shot baseline across the MPH group due to task features (e.g., eye-hand coordination, attention, encoding, and memory of spatial information) [5] and because MPH enhances task-related cognitive functions [20].

## Methods

### Sample

The sample consisted of 20 healthy, novice soldiers (mean age: 19 ± 1 yrs) who were both right handed and right-eye dominant as defined in the Edinburgh inventory [34]. All subjects had normal or corrected-to-normal vision (i.e., 20/20). Inclusion criteria were: absence of mental or physical impairments and no history of psychoactive or psychotropic substance use (screened by a previous anamnesis and a clinical examination). Moreover, those subjects had not had less than 6 hours of uninterrupted sleep on the night prior to the experiment and no previous experience in the task. All subjects signed a consent form and were aware of the experimental protocol before participation commenced. The experiment was approved by the Ethics Committee of the Federal University of Rio de Janeiro.

### Experimental procedures

Participants were equally distributed in control (CG; n = 10) and MPH (MG; n = 10; 10 mg/subject) groups using a randomized, double-blind design. Then, participants were subjected to measurement in each of three phases: Baseline (BL), Practice Blocks (PB), and Post Task Moment (PTM). The first phase included baseline resting EEG acquisition with eyes open before (BL_1_) and one hour after (BL_2_) placebo or MPH ingestion. Alpha power was examined in BL phases in order to determine whether electrophysiological differences existed between groups prior to task engagement. For example, we first tested for non-task related differences in cortical dynamics while the subjects were at rest.

The second measurement phase consisted of 4 blocks of 10 practice trials (PB_1 _to PB_4_). EEG was recorded continuously while participants performed shooting trials in a sound and light-attenuated room on an Indoor Shooting Stand. All participants sat in a comfortable chair placed immediately behind a line 5 m away from the target and a table was provided to support the pistol (Taurus PT-380) while aiming. This was done to reduce EEG muscle artifacts that are associated with weapon stabilization. In addition, participants wore special glasses that allowed occlusion to the non-dominant eye to minimize facial fatigue and double vision during aiming and as another precaution to minimize EEG artifact. We instructed each subject to shoot at the center of the target during aiming over a 3-s preparatory aiming period preceding the time of each trigger pull. All shot data were recorded by a Sam Trainer system (Knestel Elektronik, Germany), which is an electronic register device that emits infrared signals by an optical device adapted below the weapon's barrel to reflect off the target. The target consisted of a concentric ring (15.5 cm in diameter with 7.75 of radius and 0.775 mm for each impact area (i.e., 10 impact areas).

This system provides continuous weapon tracking in horizontal and vertical planes of the weapon aim point with respect to the center of the target and enabled quantification of weapon movements during aiming over a 3-s preparatory period preceding the time of each trigger pull. In this manner, it was possible to determine not only target hit locations but also the process leading to the results of each shot, such as the variability of weapon movements. Accuracy scores were determined based on which ring of the concentric circle was hit; scores ranged from 0 (missed target) to 10 (bulls eye) in increments of 1 unit. Values equal or lower than 6 were considered as low precision, between 6 and 9 were considered as moderate precision and above 9 were considered as high precision. At the shot moment, an electronic sensor adapted to the weapon transmitted an electric pulse to the recording channels of a Braintech 3000 (Emsa - Medical Instruments, Brazil). In this manner, it was possible to precisely time-lock the trigger pull of each shot in order to investigate cortical dynamics, weapon kinematics, and shooting results. The third and final phase, post-task moment (PTM), was performed two minutes after the last practice block in order to investigate electrophysiological differences existed between groups after task engagement.

### Data acquisition

EEG was recorded from 20 electrodes arranged according to the 10-20 system [35] in a nylon cap (ElectroCap Inc, Fairfax, VA, USA) yielding monopolar derivations referred to linked earlobes. In addition, two 9-mm diameter electrodes were attached above and on the external corner of the right eye, in a bipolar electrode montage, for eye-movement (EOG) artifact monitoring. Impedance of EEG and EOG electrodes was kept between 5-10 KΩ. The data acquired had total amplitude of less than 100 μV. The EEG signal was amplified with a gain of 22,000, analogically filtered between 0.01 Hz (high-pass) and 100 Hz (low-pass), and sampled at 240 Hz. Data Acquisition software (Delphi 5.0), developed at the Brain Mapping and Sensory Motor Integration Lab, was employed with the following digital filters: notch (60 Hz), high-pass of 0.3 Hz and low-pass of 25 Hz.

### Data processing and analysis

To quantify reference-free data, a visual inspection and the independent component analysis (ICA) were applied to identify and remove any remaining artifacts (e.g, eye blinks and ocular movements). Data from individual electrodes exhibiting loss of contact with the scalp or high impedances (>10 kΩ) and data from single-trial epochs exhibiting excessive movement artifact (±100 μV) were deleted. ICA is an information maximization algorithm that derives spatial filters by blind source separation of EEG signals into temporally independent and spatially-fixed components [36,37]. After artifacts were automatically removed, the remaining components were then back-projected onto the scalp electrodes by multiplying the input data by the inverse matrix of the spatial filter coefficients derived from ICA using established procedures [36]. The ICA-filtered data were then reinspected for residual artifacts using the same rejection criteria described above. A classic estimator was applied for the power spectral density or directly from the square modulus of the Fourier transform, which was performed by MATLAB 5.3 (Matworks, Inc.). Quantitative EEG parameters were extracted from the 3 s prior to trigger pull for consecutive (non-overlapping) artifact-free EEG epochs (spectral resolution: 0.25 Hz) with rectangular windowing. In this manner, based on artifact-free EEG epochs, the threshold was defined by mean plus three standard deviations and epochs with total power higher than this threshold were not integrated in the analysis.

### Spatial localization and Frequency band

We analyzed electrodes T3, T4, P3, and P4. The T3 and T4 electrodes represent anterior-temporal areas that are influenced by the somatosensory cortex, which plays an important role in supplying multimodal sensory information to performance of voluntary movements and sensorimotor integration [38]. The electrodes P3 and P4 monitor the parietal lobe, which is functionally related to integration of sensory information from different modalities and plays important roles in integrating sensory information from various parts of the body [33], in the manipulation of objects, attention, and in visuospatial processing (i.e, spatial sense and navigation) [39,40]. The alpha band (8 - 12 Hz) was chosen due to its association with perceptual, cognitive and motor mechanisms, particularly in temporal [6,7,41] and parietal regions [42].

### Statistical analysis

In relation to behavioral data, a two-way ANOVA with repeated measures was used to analyze both groups (placebo and methylphenidate) and all practice blocks (PB_1_, PB_2_, PB_3_, PB_4_). A Scheffé test was applied to analyze changes over time with practice (we deemed p < 0.05 to be significant for all statistical analyses). In addition, we used a one-way ANOVA to compare the practice blocks in every condition. Lastly, we used an independent t-test to inspect differences between groups in every block.

Absolute alpha power values were log_10_-transformed by SPSS (Statistical Package for the Social Sciences) software (version 16.0) to approximate normal distribution. A two-way ANOVA with repeated measures was used to analyze both groups (between subjects factors) and all phases, i.e., BL_1_, BL_2_, PB_1_, PB_2_, PB_3_, PB_4_, and PTM (within subjects factors) for electrodes T3, T4, P3, and P4 separately. A Scheffé test was applied to analyze significant differences between phases for each group. In addition, we used an independent t-test to verify differences between the groups at each phase.

## Results

### Behavioral measures

**Figure 1:**
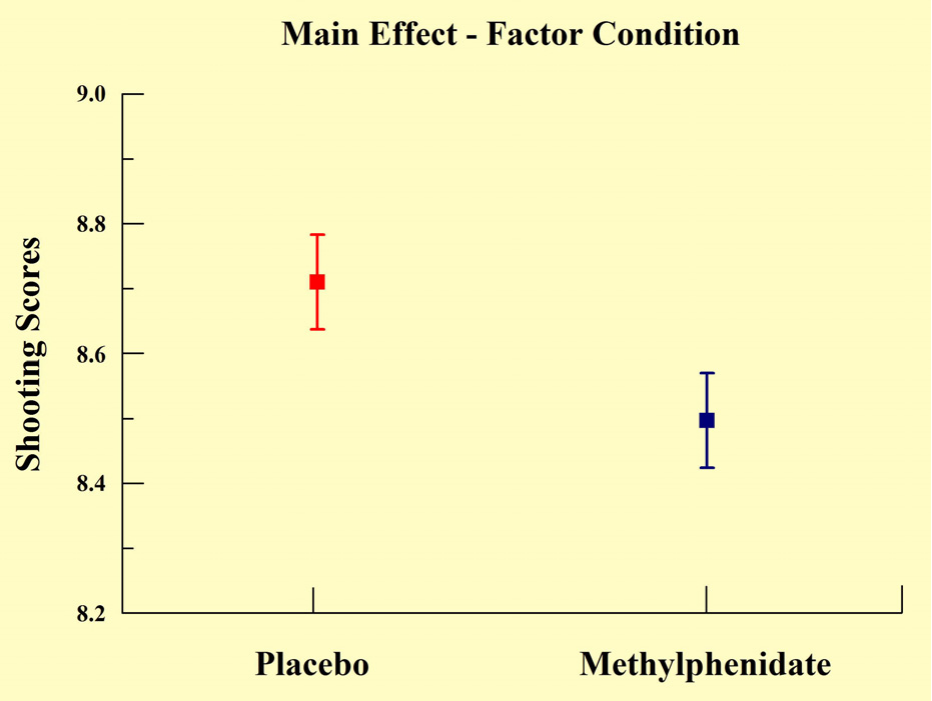
**Main effect for factor condition observed through mean and SD (p < 0.041)**.

**Figure 2:**
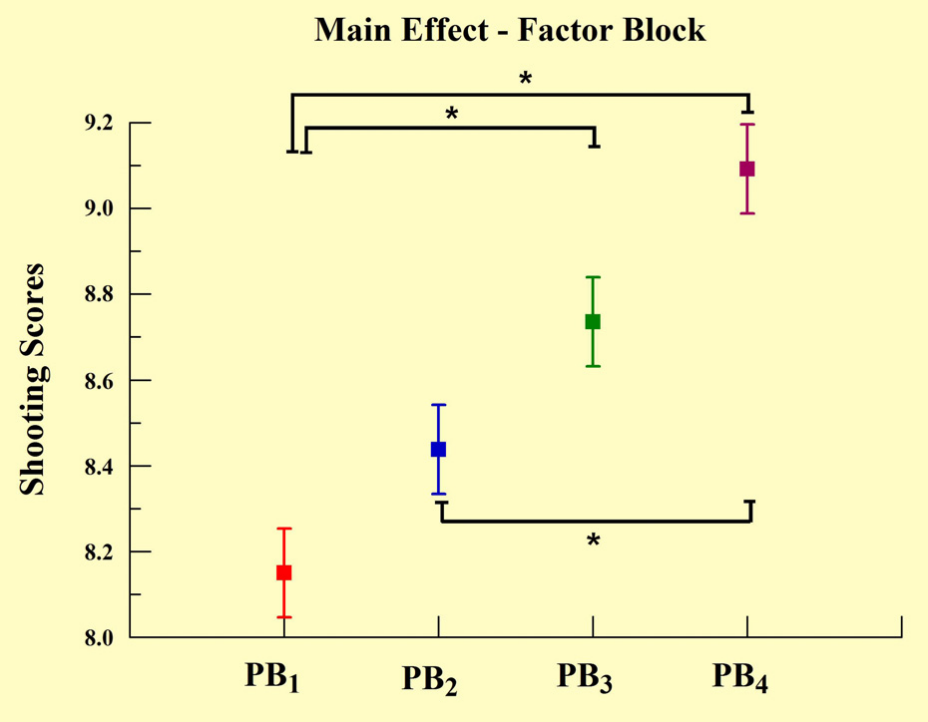
**Main effect for factor block observed through mean and SD**. *Significant difference (Scheffé test; p < 0.001).

**Figure 3:**
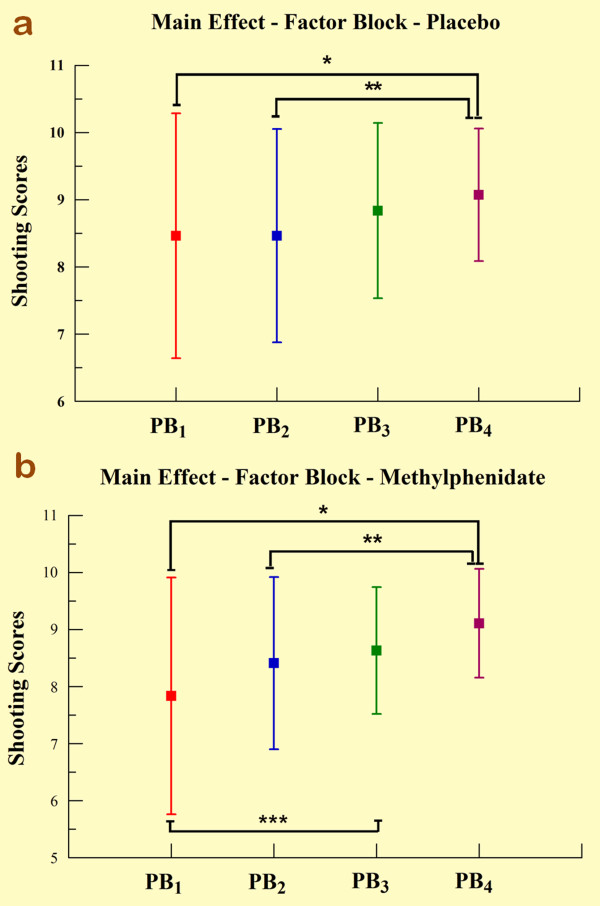
**Comparison between placebo and methylphenidate main effects for factor block.** (A) Main effect for factor block observed through mean and SD only into placebo. *Significant difference (Scheffé test; p < 0.05). **Significant difference (Scheffé test; p < 0.035). (B) Main effect for factor block observed through mean and SD only into methylphenidate. *Significant difference (Scheffé test; p < 0.003). **Significant difference (Scheffé test; p < 0.001). ***Significant difference (Scheffé test; p < 0.011).

**Figure 4:**
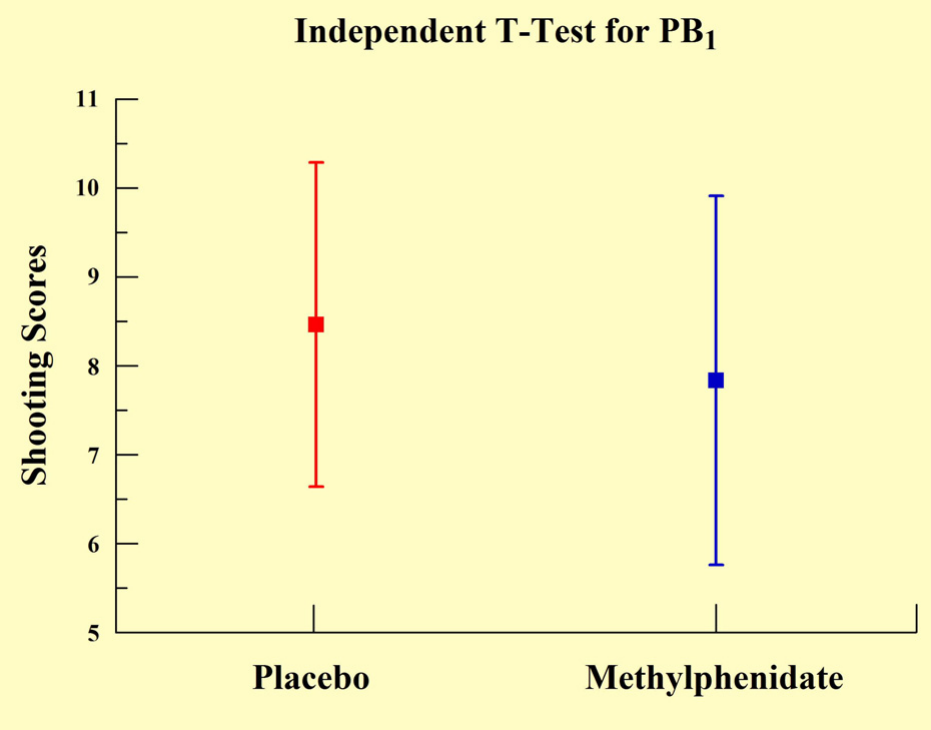
**Independent t-test between experimental conditions for the first practice block (p < 0.012)**.

The two-way ANOVA demonstrated effects for group (p = 0.041) and practice block (p < 0.001). In relation to the group main effect, the MPH group demonstrated significantly poorer shooting performance when compared with the control (see Table 1 and Figure 1). Regarding the practice block main effect, the Scheffé test revealed a significant increase in the scores over practice blocks, most evident between PB_1 _and PB_3 _(p < 0.001), PB_1 _and PB_4 _(p < 0.001), and between PB_2 _and PB_4 _(p < 0.001) (see Figure 2). The one-way ANOVA demonstrated differences between blocks for both groups. In relation to the control group, we found significant increases in shooting performance over practice blocks, most significantly from PB_1 _to PB_4 _(p < 0.05) and from PB_2 _to PB_4 _(p = 0.035) as observed in Figure 3a. We also found differences in the MPH group over practice blocks. We observed significant increases over practice blocks, most significantly from PB_1 _to PB_3 _(p = 0.003), PB_1 _to PB_4 _(p < 0.001) and PB_2 _to PB_4 _(p = 0.011) as noted in figure 3b. The independent t-test (Figure 4) demonstrated a significant difference between groups only in PB_1 _(p = 0.012).

**Table 1:** Mean and standard error of shooting performance during practice blocks.

	Placebo		Methylphenidate	
	
Blocks	*mean*	*SE*	*mean*	*SE*
**PB**_**1**_	8.464	1.8243	7.836	2.0756
**PB**_**2**_	8.465	1.5870	8.411	1.5093
**PB**_**3**_	8.838	1.3051	8.632	1.1129
**PB**_**4**_	9.074	0.9875	9.110	0.9548
**Total**	8.710	1.4774	8.497	1.5420

### Electroencephalographic measures

**Figure 5:**
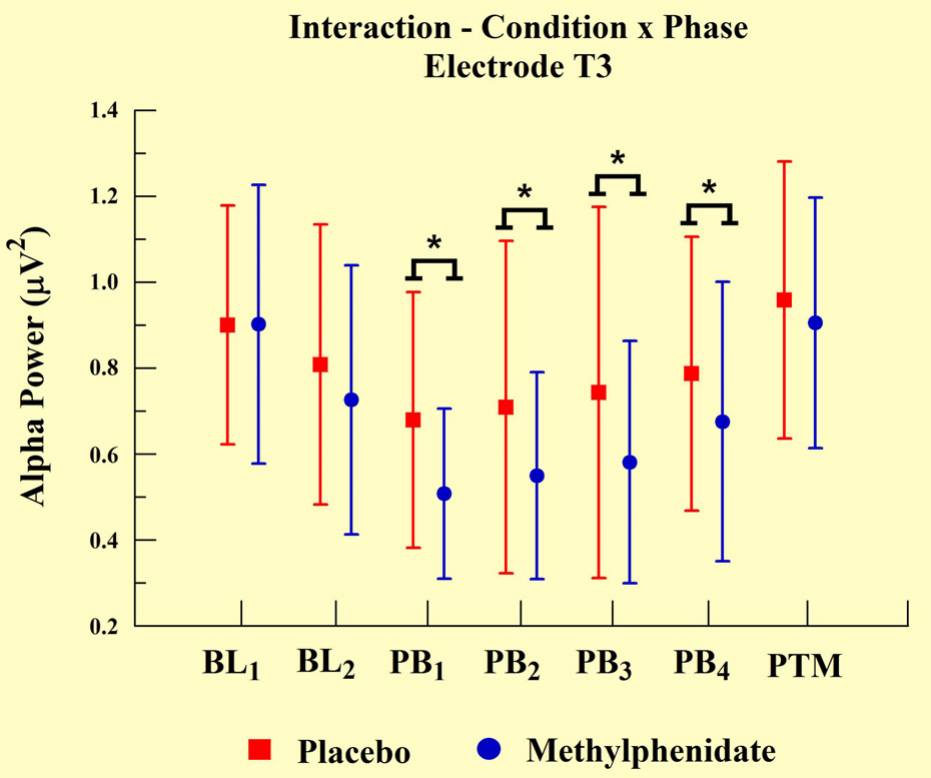
**Interaction between condition and phase factors observed through mean and SD**. *Significant difference (t-test; p < 0.001).

**Figure 6:**
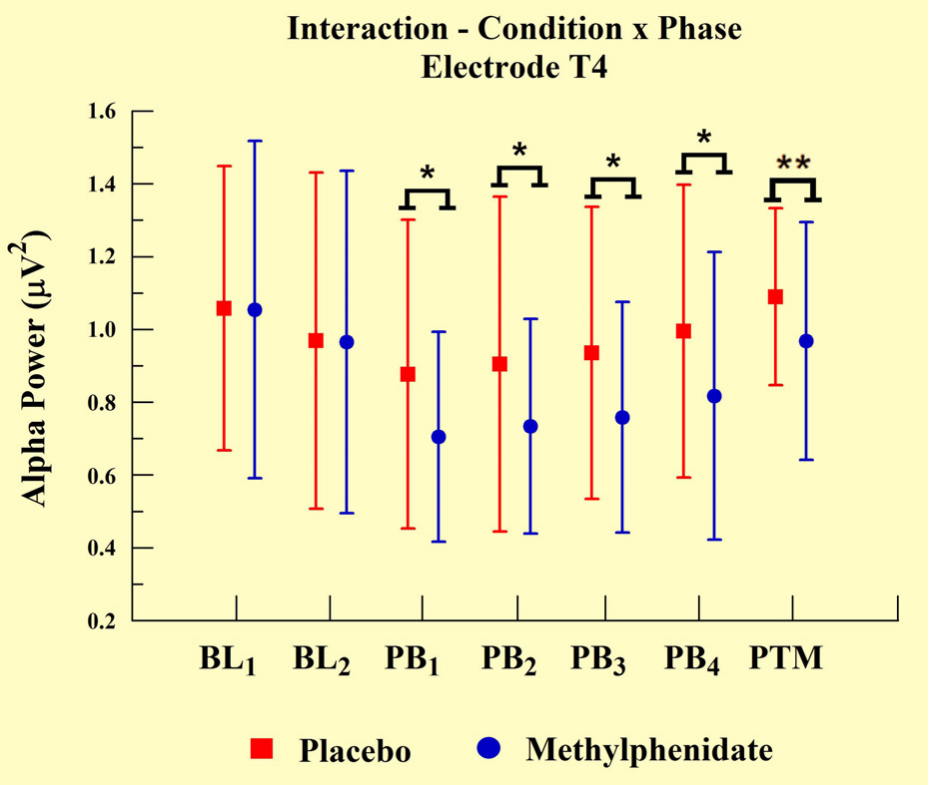
**Interaction between condition and phase factors observed through mean and SD**. *Significant difference (t-test; p < 0.001). **Significant difference (t-test; p < 0.003).

**Figure 7:**
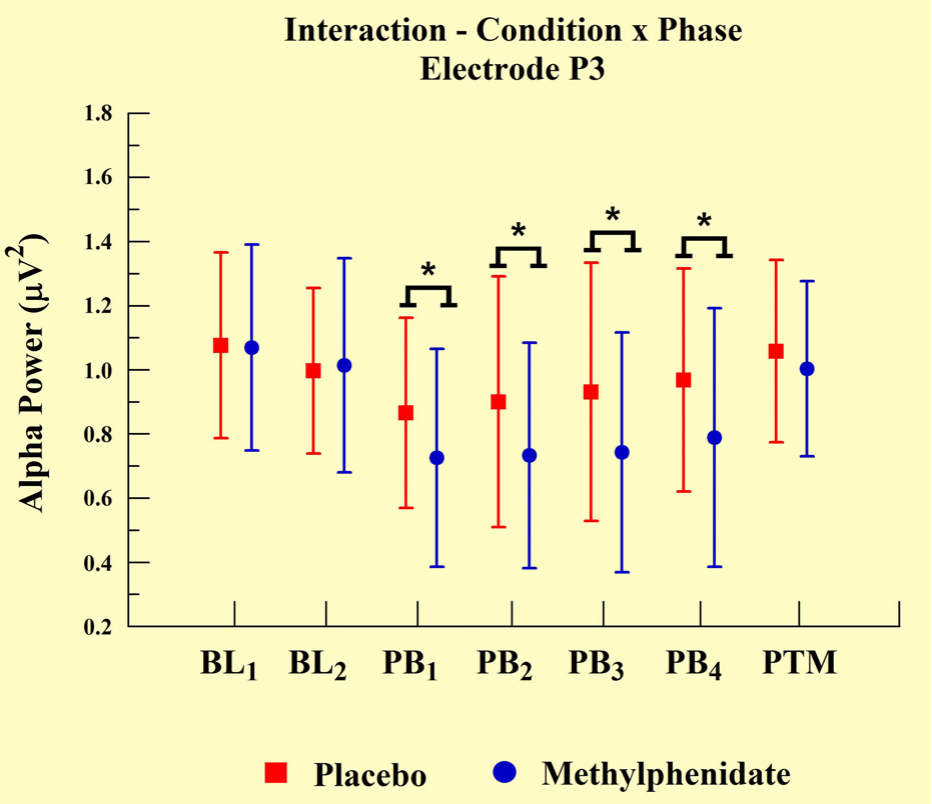
**Interaction between condition and phase factors observed through mean and SD**. *Significant difference (t-test; p < 0.001).

**Figure 8:**
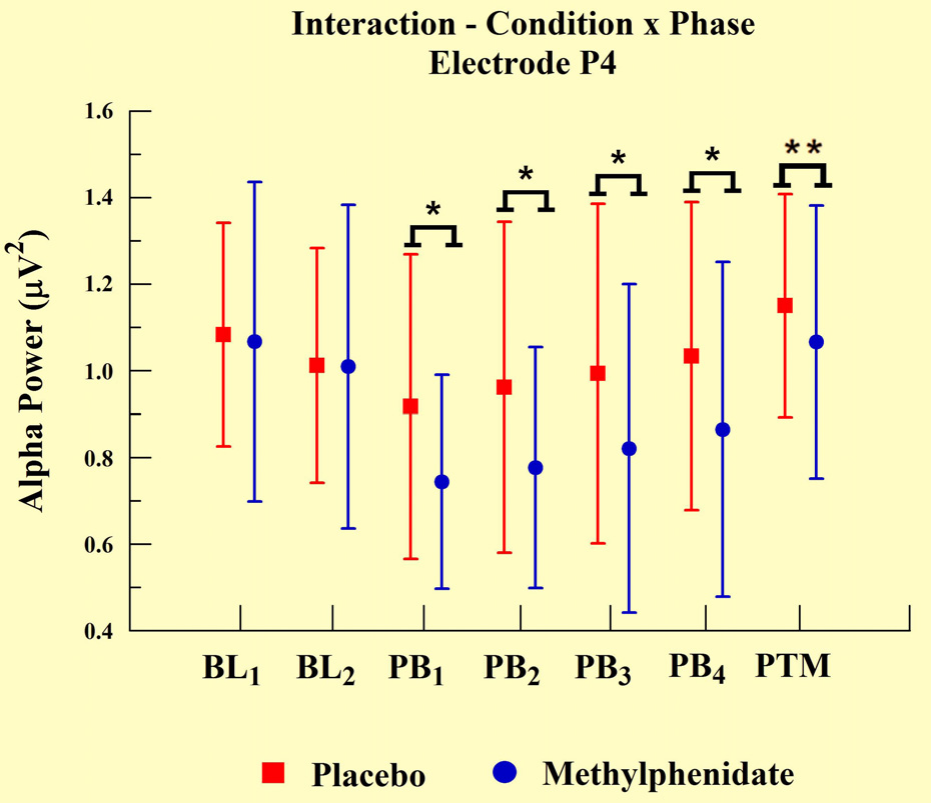
**Interaction between condition and phase factors observed through mean and SD**. *Significant difference (t-test; p < 0.001). **Significant difference (t-test; p < 0.041).

The statistical analyses demonstrated an interaction between groups and phases for electrodes T3 (p = 0.048), T4 (p = 0.046), P3 (p = 0.003), and P4 (p < 0.01). We observed a significant increase in alpha power over practice blocks, but alpha power was significantly lower for the MPH group when compared with the placebo group, as demonstrated by independent t-test (p < 0.001). However, MPH and placebo groups did not differ at other experimental phases (e.g., BL and PTM). We observed such findings only in electrodes T3 (see Figure 5) and P3 (Figure 7). In relation to the electrodes T4 (Figure 6) and P4 (see Figure 8), besides the significant decrease in alpha power during all practice blocks (p < 0.001), during the PTM we found another significant decrease in alpha power (electrode T4, p < 0.003 and electrode P4, p < 0.041) for MPH when compared with control. However, MPH and control cortical activity did not differ during BL.

## Discussion

The aim of this study was to examine the effects of MPH on absolute alpha power in the left and right temporal and parietal sites in novice shooters during the preparatory aiming period prior to trigger pull. We found main effects for group and practice blocks on behavioral measures, and interactions between group and phases on electroencephalographic measures for the electrodes T3, T4, P3 and P4. Regarding the behavioral measures, the MPH group demonstrated significantly poorer in shooting performance when compared with the control and, in addition, significant increases in the scores over practice blocks were found on both groups. In addition, regarding the electroencephalographic data, we observed a significant increase in alpha power over practice blocks, but alpha power was significantly lower for the MPH group when compared with the placebo group. Moreover, we observed a significant decrease in alpha power in electrodes T4 and P4 during PTM. Traditionally, alpha band oscillations are produced in widespread cortical areas and modulated through thalamocortical and corticocortical connections [27-29]. Practice-related increases in alpha power might be interpreted as indicative of a gradual decrease in cortical neuron activation during task performance, such as after skill development. Our interest in the temporal and parietal lobe follows from several studies that reported the involvement of the temporal lobe in transmission of multimodal sensory information from the posterior superior temporal sulcus to the parietal lobe [31,32]. The parietal lobe comprises somatosensory cortex and the dorsal stream of the visual system, which enables regions of the parietal cortex to map objects perceived visually into body coordinate positions [39].

We expected an evident higher bi-hemispheric cortical activation (e.g., decrease in alpha power), in the preparatory period, relative to pre-shot baseline across MPH group in contrast to the control group due to task features (eye-hand coordination, attention, encoding, and memory of spatial information) because MPH enhances cognitive functions related to the task. Such activation would decrease, as shown by an increase in alpha power, differently between the groups across successive task practice, according to cognitive strategies undertaken in the brain. In this manner, the assessment of qEEG may unveil how the cerebral cortex participates in the organization and integration of sensory information and, thus, the performance of cognitive operations and achievement of motor control during the performance of multiple complex tasks under MPH effects.

### Behavioral measures

We found a group main effect and we observed that performance of the MPH group was statistically lower on shooting accuracy when compared with the control group. Moreover, we found a practice block main effect and verified a learning effect within both groups that improved performance progressively throughout the task. Examining the main effect in detail from the comparison of two groups for each block, we observed that such difference of performance is most pronounced in PB_1_. This finding suggests that the action of MPH may demand an initial readjustment period of integrated operations relative to the sensorimotor system. In other words, the MPH group underwent a period of initial instability due to a possible modulation in neural activities [43]. However, after the end of the PB_1_, a new stabilization occurred among neural circuits during the task and the MPH group performance equated with control group performance during the subsequent practice blocks, eventually exceeding it in the last block. However, this effect did not reach statistical significance (p = 0.052).

We applied a one-way ANOVA to investigate possible differences among PBs for each group. First, we observed that MPH modified learning process rhythm. Whereas in the control group, significant differences on performance among the PBs only occurred on the last block (PB_4 _>PB_1 _and PB_4 _>PB_2_), in the MPH group these differences were detected in the third block (PB_3 _>PB_1_; PB_4 _>PB_1_; and PB_4 _>PB_2_). Therefore, it seems that during performance of the task, MPH group had an apparent reduction in performance, perhaps sensorimotor instability, in PB_1 _and that improvement of performance (increase in accuracy) was progressively achieved similarly in the control group. Our results show a best, but statistically non-significant, MPH group performance on the last block, which led us to the following conclusion: if we 1) increased the number of shots on every PB, 2) increased the number of PBs, or 3) gave a larger dose of the drug, MPH group performance could continue increasing in relation to the control group, possibly achieving statistical significance. To test this hypothesis, further experiments must be conducted.

Our results demonstrate an improvement in subjects' performance along the practice blocks for both groups, most evident between PB_1 _and PB_4 _and between PB_2 _and PB_4_. In early stages of learning the dependence of sensory stimuli is essential to task execution. Subjects had to coordinate the basic skills of the task, such as shot position, grip arm, and breath, during the preparatory aiming period prior to trigger pull. Such factors explain the lower accuracy in shot performance in PB_1 _and PB_2_. On the contrary, in PB_3 _and PB_4_, subjects demonstrated an increase in basic skills performance and were able to focus attention on main skills. As a result of practice, fine adjustments made by the CNS in order to integrate visual and somatosensory stimuli [7] allowed shooters to achieve higher scores.

In more advanced stages of learning, subjects better understand the rules and strategies of the task, and can automatically perform most basic skills, reducing task complexity [42]. Consequently, shooters could selectively focus their attention on the shot demands, such as integrating the precise time of trigger pull with the continuous flow of visual and proprioceptive feedback during aiming, even intentionally minimizing the necessity of separately regulating each task component. The final result was a refinement of the perceptual-motor process, which led to improvement of movement accuracy and quality [44]. In line with this observation, our results indicate that MPH in initial stages of learning promotes a sensorimotor instability during task performance, as observed during PB_1_, and later improved performance (increase in accuracy).

### EEG measures

Our findings indicate significantly lower levels of absolute alpha power values in MPH group compared to control group. According to previous findings, alpha amplitude is inversely related to cortical network activation. These data suggest an augmentation of cortical activation in subjects induced by MPH [26-28]. This increase was verified through electrodes T3, T4, P3, and P4. The performance of practical shooting requires the subject to manipulate a great deal of sensory information to achieve an appropriate performance. Especially, the increase of cortical activity in temporal and parietal regions can be clarified by the involvement of these areas in this type of task. The temporal region is involved with the integration of sensory information and the formation of new memories of a spatial nature [8]. Conversely, the parietal region is involved with the formation and follow-up of spatial coordinates among limbs and environmental objects [45]. Within this context, our finding demonstrated that temporal and parietal cortices are susceptible to MPH action.

The differences in alpha absolute power values between MPH and control groups were observed in all electrodes, and were associated with motor task execution. The absence of a difference between groups in BL_2_ (one hour after placebo or drug ingestion) and the difference observed in every PB and in PTM, suggested that the drug's modulatory action became apparent only when cognitive and psychomotor processes were involved. These findings indicate that spontaneous qEEG analyses, in the absence of a specific task engagement, might be a less effective strategy to observe MPH behavior. Regarding the difference of absolute power in PTM, this presented a more focal topographic pattern, detected only on both electrodes of the right hemisphere (T4 and P4). We interpret this last finding as an indicator that task practice stimulated automaticity of the training procedure, putting special emphasis on spatial demands to adapt hand position, i.e. aiming the gun at the center of the target. The right cerebral hemisphere plays a dominant role in spatial function [6,45,46], and may explain why absolute power values remained altered in the MPH group after the task ended (during PTM). In other words, neural networks of these cortical areas possibly presented long-term potentialization in terms of electrical activity, expressing a set of sensorimotor learned elements.

Regarding left hemisphere electrodes (T3 and P3), our findings indicate a reduction in alpha absolute power in the MPH group compared to the control group during the PBs. However, this was not limited to PTM, which was the case for the right hemisphere electrodes. In line with this observation, EEG is sensitive in identifying changes produced by a drug's action, particularly on active brain dynamics, thereby suggesting that the contribution of the left temporal and parietal regions occurred only during task execution. A possible explanation for this relates to the role of the left hemisphere in motor function [45], which would not be needed after task completion. This agrees with other reports on features of brain dominance obtained by neuroimaging techniques [47,48].

Our last finding is that an increase in alpha absolute power observed from the first movement to the last PB in all electrodes occurred in both MPH and control groups. According to other studies, the increase in alpha power indicates a reduction in information load necessary for brain integration and processing to efficiently meet motor task demands [49]. In other words, with practice the CNS develops strategies that become more efficient in sensorimotor processing [50]. In this case, activation of the least number of neural populations with the lowest intensity is used to perform a task successfully. Several reports have demonstrated that a continuous learning process occurs throughout the activation of cortical areas that are predominantly involved in cognition (for review see [45]). In line with this, cortical areas handling motor and spatial activity linked to the task features play a prominent role (for review see [5]).

In conclusion, despite the observation that there is no interaction between behavioral and EEG data, our findings suggest that MPH does not prevent the learning process of the task in healthy subjects. However, during the PBs MPH did not favor performance when compared with the control group. It seems that CNS MPH action possibly demands an initial readjustment period of integrated operations relative to the sensorimotor system. In other words, MPH seems to provoke a period of initial instability due to a possible modulation in neural activity, which can be visualized by lower levels of alpha power (higher cortical activity). However, after the end of the PB_1_ a new stabilization occurs among neural circuits during the task, due to the practice of the task and its features, resulting in lower levels of alpha power (higher cortical activity) toward the end of the task. Thus, MPH group performance equated gradually with control group performance and at last exceeded it, albeit without achieving statistical significance. We suggest that further studies be conducted using this paradigm to investigate methylphenidate action, however, with an increased volume of practice as well as a higher dose of MPH.

## Competing interests

The authors declare that they have no competing interests.

## Authors' contributions

FP, SM, OAC, CAD, ST, BV, PR and AEN designed, conducted the literature review and drafted most of the manuscript. MC, DM, LFHB, HB, MC, RP, SK, MMG, SDS, BAN performed the literature review and the drafting of the manuscript. All authors were equally involved in reading and approving the final manuscript.
